# Substance Use and Dual Diagnosis Disorders: Future Epidemiology, Determinants, and Policies

**DOI:** 10.1155/2015/145905

**Published:** 2015-05-05

**Authors:** Sahoo Saddichha, Christian G. Schütz, Baxi Neeraj Prasad Sinha, Narayana Manjunatha

**Affiliations:** ^1^North Western Mental Health, Melbourne Health, Melbourne, Australia; ^2^Department of Psychiatry, University of British Columbia, Vancouver, Canada; ^3^Affective Disorders Team, Wessex House, Stockton-on-Tees, UK; ^4^Department of Psychiatry, National Institute of Mental Health & Neurosciences, Bangalore, India

Dual diagnosis or dual disorders are terms used to define the presence of an addictive disorder and another mental health disorder in an individual. With a high prevalence of dual diagnosis (above 50%) being well documented in clinical and epidemiological studies, clinicians, researchers, and policy makers have increasingly been paying attention to the challenges of identifying and implementing adequate management of cooccurring disorders/dual pathology, especially since they have been frequently associated with relapses, poor treatment engagement, and overall unsatisfactory treatment outcomes. The papers selected for this special issue represent a good panel for addressing this challenge. It is however quite certain that the subject is extremely vast and, hence, the selected topic and the papers are not an exhaustive representation of this area of dual diagnostic disorders. Nonetheless, they represent the rich and complex web of interactions which underplay dual diagnosis, which we have the pleasure of sharing with the readers.

The special issue contains five papers, from different geographical regions of the world, each dealing with a different subject within this vast area. A paper from Germany titled “Reflections on Addiction in Students Using Stimulants for Neuroenhancement: A Preliminary Interview Study” uses face to face interviews with university students to explore determinants of nonmedicinal uses of methylphenidate and amphetamines for pharmacological neuroenhancement. While highlighting the need for long term empirical research, on the basis of some quite interesting results, the authors conclude that the beliefs and behavior of their sample population appear to be risky in terms of development of addiction. Such findings can help in understanding the underpinnings of addictions in student population and may have the potential to ultimately improve targeted interventions for this important group.

In a paper from Malaysia titled “The Effect of Nicotine Dependence on Psychopathology in Patients with Schizophrenia,” A. Yee et al. study the prevalence of nicotine dependence in a cross-sectional study and investigate the effects of nicotine dependence on psychopathology among 180 outpatients with schizophrenia at a general hospital in Malaysia. They have observed a higher prevalence of nicotine dependence among patients with schizophrenia when compared to the general population in Malaysia. They have also observed a significant association between negative symptoms of schizophrenia and nicotine use, which supported the notion of self-medication hypothesis of schizophrenia.

In another paper from Brazil titled “Revictimization of Violence Suffered by Those Diagnosed with Alcohol Dependence in the General Population,” F. G. Moreira et al. studied the vicious association of violence and alcohol dependence syndrome in a general population. Although they observed that urban and familial violence in the general population and alcohol dependence had a complex interplay, they felt that a policy of reducing familial and domestic violence may be necessary to reduce alcohol dependence. This is an interesting conclusion and calls for further validation in well-designed longitudinal studies.

In a paper from Australia titled “Khat Use: What Is the Problem and What Can Be Done?” which was unique in being a qualitative research study as well as studying a minority population, Y. S. Omar et al. used the techniques of focused group discussions and thematic analysis to address khat use and evolve strategies to deal with this rapidly progressing problem.

In a paper from Canada, titled “Attention Deficit Hyperactivity Disorder Symptoms, Comorbidities, Substance Use, and Social Outcomes among Men and Women in a Canadian Sample,” E. Vingilis et al. screened for ADHD symptoms and its correlates including substance use. They observed a higher lifetime cocaine use and comorbid anxiety and depression, which points to the difficulties of being able to manage this complex group of patients.

